# The change in Ig regulation from children to adults disconnects the correlation with the 3′RR hs1.2 polymorphism

**DOI:** 10.1186/s12865-014-0045-0

**Published:** 2014-11-13

**Authors:** Eliseo Serone, Cristina Daleno, Nicola Principi, Laura Porretti, Valentina Iacoacci, Cesare Gargioli, Andrea Magrini, Renato Massoud, Pietro D’Addabbo, Marco Cattalini, Vincenzo Giambra, Alessandro Plebani, Susanna Esposito, Domenico Frezza

**Affiliations:** Department of Life, Health and Environmental Sciences, University of L’Aquila, L’Aquila, Italy; Department of Biology “Enrico Calef”, University of Roma Tor Vergata, 00133 Rome, Italy; Department of Pathophysiology and Transplantation, Università degli Studi di Milano, Fondazione IRCCS Ca’ Granda Ospedale Maggiore Policlinico, Milan, Italy; Department of Sperimental Medicine, Policlinico Tor Vergata, Rome, Italy; Department of Biology, University of Bari, Bari, Italy; Terry Fox Laboratory, British Columbia Cancer Research Centre, Vancouver, BC Canada; Pediatrics Clinic, Department of Clinical and Experimental Sciences, Università di Brescia e Spedali Civili di Brescia, Brescia, Italy

**Keywords:** Genotyping, B cell markers, Immunoglobulin heavy chain, Enhancer hs1.2, Immune system regulation, NF-κB, SP1, Transcription factor consensus, Aging

## Abstract

**Background:**

In the immune system, the serum levels of immunoglobulin (Ig) increase gradually during ageing. Through B cell development, the Ig heavy chain expression is modulated by a regulatory region at the 3’ of the constant alpha gene (3’RR), in single copy in rodents and, due to a large duplication, in two copies in apes. The human 3’RR1 and 3’RR2 are both characterized by three enhancers, the central of which, namely hs1.2, is highly polymorphic. Human hs1.2 has four different variants with unique binding sites for transcription factors (e.g. NF-kB and SP1) and shows variable allelic frequencies in populations with immune disorders. In previous works, we have reported that in several autoimmune diseases the *2 allele of hs1.2 is genetically associated to high level of IgM in peripheral blood. In subjects with altered levels of circulating Ig, an increased level was associated to *2 allele of hs1.2 and low levels corresponded to high frequency of *1 allele.

During ageing there is a physiological increase of Ig concentrations in the serum. Therefore, for this study, we hypothesized that the hs1.2 variants may impact differently the levels of secreted Ig during the growth.

**Results:**

We have correlated the allelic frequencies of hs1.2 with IgM, IgG and IgA serum concentrations in two cohorts of healthy people of different age and after three years follow-up in children homozygous for the allele. Here we show that when the expression levels of Ig in children are low and medium, the frequencies of *1 and *2 alleles are the same. Instead, when the Ig expression levels are high, there is a significantly higher frequency of the allele *2. The follow-up of children homozygous for *1 and *2 alleles showed that the increase or decrease of circulating Ig was not dependent on the number of circulating mature B cells.

**Conclusions:**

These data support the idea that under physiologic condition there is a switch of regulative pathways involved in the maturation of Ig during ageing. This mechanism is evidenced by hs1.2 variants that in children but not in adults participate to Ig production, coordinating the three class levels.

**Electronic supplementary material:**

The online version of this article (doi:10.1186/s12865-014-0045-0) contains supplementary material, which is available to authorized users.

## Background

The serum levels of Immunoglobulins (Ig) are the result of regulated processes involving B cell development and the progressive expression of immunoglobulin heavy chain (*Igh*) genes [1]. During B cell differentiation, IgH locus (14q32.33) undergoes various DNA rearrangements and epigenetic changes, necessary for the generation of antibody repertoire [2-5].

**Figure 1:**
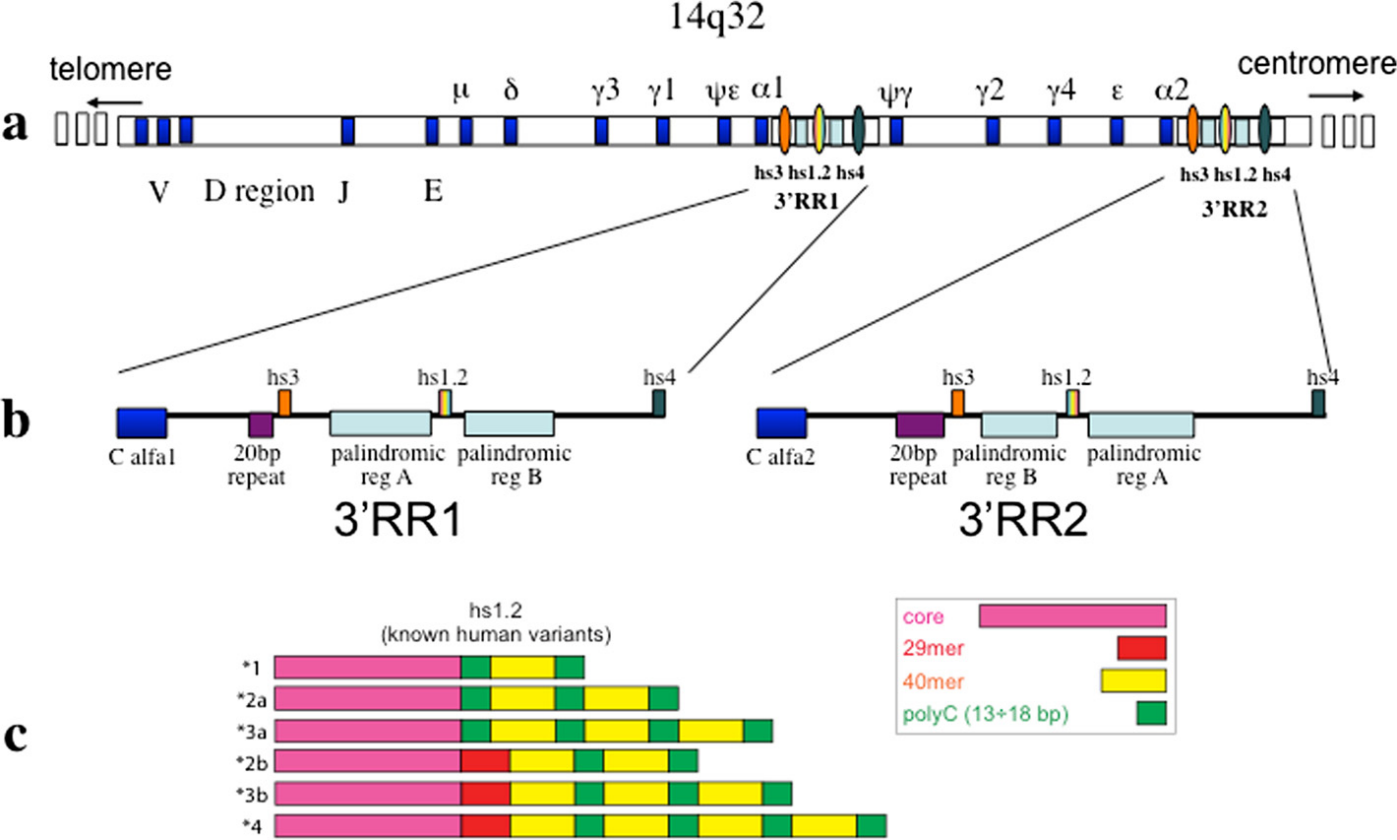
**hs1.2 location and known human variants. a)** The locus of the Ig heavy chain with the variable, constant and regulatory elements. The three enhancers of 3′RR1 and 3′RR2 are conserved in order: hs3 (orange), hs1.2 (rainbow), hs4 (dark green). Nevertheless, hs1.2 sequence is inverted in 3′RR2 with respect to the 3′RR1. **b)** Regulatory regions at the 3′ of the constant alpha1 and alpha2 genes (blue). A 20 bp conserved repeats is shown in violet, while the palindromic regions in light blue. **c)** Scheme of the six variants of the enhancer hs1.2 known in human (see included caption box for colors explanation).

The regulatory region 3′RR at the 3′ of the constant alpha gene is in single copy in rodents and in two copies in apes due to a large duplication described in Figure 1 [6].

The human IgH locus is characterized by two 3′ regulatory regions (3′RR) caused by a duplication, both downstream with respect to heavy chain alpha (Cα) genes (see Figure 1). Each 3′RR is composed of three different enhancers. In both cases the central enhancer hs1.2 is located in one region with a palindromic sequence conserved in structure but not in sequence [7-9] and contains a 40-bp tandem-repeat DNA motif, polymorphic for number of copies and conserved in different species of mammals [10,11]. In humans this 40 bp region is repeated from one to four times. The hs1.2 allele with two copies of 40 bp element is called allele *2 and carries a unique binding site for NF-κB transcription factor [12]. In the machinery of the Ig maturation the class switches when IgG2, IgG4, IgA2 and IgE are produced. Since 3′RR1 is deleted along with the rest of the heavy chain involved in the excision, the switched allele will be under the control of the 3′RR2. Of note, more then 95% of human subjects bear *4 allele of hs1.2 in 3′RR2 giving a high homogeneity of the expression and not the variability observed for 3′RR1 (see Figure 1). Different selective advantages or disadvantages are related to possible different functions. Hs1.2 allele *2 in the distal 3′RR1 is significantly more frequent in patients with several autoimmune diseases, such as celiac disease, psoriasis, systemic sclerosis, rheumatoid arthritis, and lupus erythematosus with respect to healthy control group [12-15], and is associated with high levels of IgM in peripheral blood [16]. Of note, patients with IgA deficiency show a significant correlation with hs1.2 allele *1 [16]. Finally, new evidence have been reported on the differences among hs1.2 polymorphic variants indicating complex interactions between binding factors and enhancers after stimulation of mouse and human B cells [17].

It has already been described that follow-up of children through adulthood shows a gradual increase of Ig concentrations in serum [1]. Therefore, in order to determine if there is a genetic correlation between hs1.2 alleles and the levels of circulating Ig in children and adults, we compared the concentration of circulating Ig in association with allelic frequency of hs1.2 enhancer in one cohort of adults and one of young healthy subjects. Our data show that the IgM, IgG and IgA serum concentrations are significantly associated with the hs1.2 alleles in youth, but not in adult population, suggesting a fundamental role of hs1.2 enhancer variants in the Ig expression in children.

## Results and discussion

One cohort of healthy children and one of adults were recruited and measured for the Ig serum levels. Individuals were divided in three groups with low, medium and high serum levels with respect to the Ig median of each cohort (Additional file 1: Table S1 and in the Methods). The Ig levels were all in the normal range. Table 1A-B shows the allelic frequencies for both cohorts within each Ig class and reports that hs1.2 alleles are associated with Ig serum levels in children, but not in adults (Additional file 2: Figure S1 dot-plots the data summarized in both Table 1 and Additional file 1: Table S1). In fact, the comparison of Low (L) vs High (H) and Medium (M) vs H serum levels of Ig shows highly significant different allelic frequencies (constantly p < 9.9E-08), only in the population of children. Similar variations of *1 and *2 allele frequencies in children were observed in each Ig class of young cohort: the *1 allele frequency decreases from the range of 41%-49% in L and M groups to the range of 2%-5% in H group and, on the contrary, *2 allele frequency increased from the range of 39%-49% in L and M groups to the range of 79%-82% in H group. It is remarkable that no young subjects homozygous for *1 allele were included in the H groups of IgM and IgG, and only one young subject was present in the H group of IgA. The frequencies of 2/2 homozygous genotype in children of L and M groups were between 1.7%-13.3%, while the H group of each Ig class was comprised between 60.0%-66.6%. In children and adults, the medians of Ig level were very similar, in agreement with the ranges reported in literature [1] (see Additional file 1: Table S1). Nevertheless, the adult cohort did not show the same level and significance of allelic frequency variations than children when subdivided for Ig levels in L, M, and H groups (Table 1A; 0.69 < p < 0.92). Consequently the probability of one subject to be in one group of Ig level and also in the same group for the other Ig classes is very high in children, but not in adults (see Additional file 1: Table S2). The genetic association between Ig concentrations and hs1.2 polymorphism suggests likely age-related changes in Ig expression for children homozygous for *1 or *2 allele. Therefore, we followed up the Ig levels of those children for three years. As reported in Table 2, after three years (from five to eight) *1 allele homozygous children showed an increase of IgG and IgA levels, while *2 allele homozygous children showed a decrease in all Ig levels. We also investigated if B cell subpopulations of peripheral blood were similar in two homozygous groups by flow cytometric analysis, using IgM and IgD surface cell markers for the distribution of naive, marginal zone, IgM memory and switched memory B cells (Additional file 1: Table S3). Children homozygous for *1 and *2 alleles show no differences in the distribution of B cell subtypes, suggesting moreover that the number of B subtypes of circulating cells does not influence IgM concentration in individual with different genotype (Additional file 1: Table S3).

**Table 1:** **Allelic frequencies of hs1.2 enhancer in children (A) and adults (B) divided for the serum levels of Ig**

**A**		**IgM**			**IgG**			**IgA**		
		**Low**	**Medium**	**High**	**Low**	**Medium**	**High**	**Low**	**Medium**	**High**
**Children (f)**	**ALLELE 1**	0.47 ± 0.03	0.44 ± 0.04	0.02 ± 0.01	0.48 ± 0.05	0.41 ± 0.04	0.05 ± 0.02	0.49 ± 0.04	0.40 ± 0.06	0.05 ± 0.03
	**ALLELE 2**	0.43 ± 0.04	0.46 ± 0.04	0.81 ± 0.04	0.44 ± 0.03	0.47 ± 0.06	0.79 ± 0.04	0.39 ± 0.05	0.49 ± 0.04	0.82 ± 0.03
	**ALLELE 3**	0	0	0	0	0	0	0	0	0
	**ALLELE 4**	0.1 ± 0.03	0.1 ± 0.02	0.17 ± 0.03	0.08 ± 0.02	0.12 ± 0.03	0.16 ± 0.03	0.12 ± 0.03	0.11 ± 0.03	0.13 ± 0.02
	**n.**	60	70	60	60	70	60	60	70	60
**B**										
**Adults (f)**	**ALLELE 1**	0.29 ± 0.03	0.32 ± 0.03	0.25 ± 0.02	0.30 ± 0.02	0.30 ± 0.02	0.26 ± 0.02	0.34 ± 0.03	0.26 ± 0.04	0.27 ± 002
	**ALLELE 2**	0.56 ± 0.04	0.54 ± 0.02	0.58 ± 0.03	0.54 ± 0.04	0.55 ± 0.06	0.58 ± 0.04	0.52 ± 0.04	0.58 ± 0.03	0.57 ± 0.07
	**ALLELE 3**	0	0.01 ± 0.01	001 ± 0.01	0.01 ± 0.01	0.01 ± 0.01	0.01 ± 0.01	0.01 ± 0.01	0	0.01 ± 0.01
	**ALLELE 4**	0.15 ± 0.01	0.13 ± 0.02	0.16 ± 0.01	0.15 ± 0,01	0.14 ± 0.01	0.16 ± 0.02	0.13 ± 0.03	0.16 ± 0.01	0.15 ± 0.02
	**n.**	110	120	110	110	120	110	110	120	110

**(A)** pValue IgM LOW vs HIGH = 3.9E-12; LOW vs MEDIUM = 8E-04; MEDIUM vs HIGH = 5.2E-10; pValue IgA LOW vs HIGH = 5.6E-11; LOW vs MEDIUM = 1E-03; MEDIUM vs HIGH = 9.9E-08; pValue IgG LOW vs HIGH = 8.3E-11; LOW vs MEDIUM = 1E-03; MEDIUM vs HIGH = 4.6E-08.

**(B)** pValue IgM LOW vs HIGH = 0.92; LOW vs MEDIUM = 0.97; MEDIUM vs HIGH = 0.79; pValue IgG LOW vs HIGH = 0.53; LOW vs MEDIUM = 0.61; MEDIUM vs HIGH = 0.99; pValue IgA LOW vs HIGH 0.14; LOW vs MEDIUM 0.11; MEDIUM vs HIGH 0.79.

(n. = Number of subjects).

**Table 2:** **Ig median follow-up from 5 to 8 years**

		**IgM median**			**IgG median**			**IgA median**		
**Genotype**	**n.**	**5 years (mg/dl)**	**8 years (mg/dl)**	**t test**	**5 years (mg/dl)**	**8 years (mg/dl)**	**t test**	**5 years (mg/dl)**	**8 years (mg/dl)**	**t test**
**1/1**	10	98.5 ± 0.07	94.5 ± 0.05	4.94E-01	880.5 ± 0.12	1029.5 ± 0.14	1.31E-01	86.5 ± 0.04	102 ± 0.06	9.86E-02
**2/2**	30	217.5 ± 0.11	103 ± 0.09	1.09E-11	1674.5 ± 0.16	979 ± 0.13	1.98E-13	208 ± 0.12	105.5 ± 0.08	1.29E-13

Follow-up of Ig concentrations in children homozygous for hs1.2 *1 and *2 alleles.

The children cohort (5 years old as median) was tested at time 0 and three years later. The t test shows a high significance for all the differences in the Ig median levels of 2/2 homozygous children, whereas the differences in 1/1 children were always not significant.

Previous evidence showed the association of Ig levels with 3′RR1 enhancer hs1.2 polymorphism in autoimmune diseases. In these experiments we studied the role of hs1.2 polymorphism in physiological conditions in healthy subjects. Our results show that in children there is a significant correlation between different hs1.2 alleles and Ig expression levels, suggesting that hs1.2 polymorphism might primarily influence the concentration of Ig classes in serum during childhood. Moreover the follow-up of the children homozygous for the *1 allele showed that, after the fifth year of age, the Ig median values for all three classes increase; instead, in subjects homozygous for the *2 allele the values strongly decrease. This trend leads to the final adult equilibrium observed where no correlation is shown with Ig levels and hs1.2 alleles.

## Conclusions

Our data imply that during physiological development there is a switch of regulatory mechanisms involved in Ig expression that might be reverted in autoimmune diseases [12-14]. The similar distribution of B cells subpopulations in homozygous children for *1 and *2 alleles (most frequent in European populations) suggests that the different Ig levels are obtained by different Ig secretion of the cells and not by the different number of B cells.

In conclusion we like assessing that there is a mechanism, correlated to hs1.2 polymorphism, that in children leads to a parallel control of IgA, IgG and IgM, while in adulthood changes to produce the three Ig classes at different concentrations.

These observations raise the possibility that in children the polymorphism of the hs1.2 enhancer is a relevant component in regulatory mechanism involved in Ig expression. The presence in Europe of higher *2 allele [18] and these results related to Ig levels in children can give an explanation for the selective advantage of the allele balanced by the disadvantage in adulthood for the association to autoimmune disease. It must be mentioned that in humans the presence of two copies of hs1.2 derived by the duplication of the 3′RR can give the possibility to the alleles of the first copy at 3′ of alpha-1 to influence the regulation otherwise absent if the gamma 2/4, epsilon and alpha-2 switch occurs.

Moreover this evidence answers the question about the function of the alleles. In fact, the positive association of *2 allele with high Ig levels in childhood could be the reason for the positive selection after the migration out of Africa: a condition of shorter life expectancy could have led to a reduced risk for immune-diseases development.

Nevertheless, further studies will be required to address the role of hs1.2 during aging.

## Methods

The cohort of healthy children was composed of 190 subjects (88 females and 102 males; age range 3–5 years; median age 3.75 years). A second cohort of 340 adult subjects consisted of 218 females (64%) and 122 males (36%) and with an age range from 19 to 33 years (median age 24 years). Research was carried out in compliance with the Helsinki Declaration and the study was approved by the Ethics Committee of Fondazione IRCCS Ca’ Granda Ospedale Maggiore Policlinico in Milan, Italy. The parents of all of the participants gave their written informed consent. From each individual, genomic DNA was extracted from blood by standard methods and a selective PCR for 3′RR1 region containing the polymorphic hs1.2 alleles was performed by conditions previously described [11]. The frequencies of four hs1.2 alleles detected in two cohorts were evaluated by standard chi-square tests. The median value of Ig concentration of each cohort was used to get an equal number of subjects with low and high Ig levels, the group with medium level contained the remaining subjects. The range of Ig levels of each sub-group resulted as the minimum and maximum value of the subjects in that sub-group as a consequence of the subdivision determined by the median of the entire cohort. The dosage of Ig is mg/dl. The subjects were then classified arbitrarily by IgM, IgG and IgA serum concentrations (Low [L], Medium [M], and High [H]) respect to the median value and by age, children (<5 y/o) and adults (>19 y/o), as shown in Table 1A-B. All the subjects had Ig values within the normal range concentrations. Finally, fifty μl of whole blood was labeled as well by 6-color TBNK™ kit reagent (BD Biosciences), according to manufacturer’s recommendations for quantification and follow up of CD19^+^ cells.

## Additional files

Additional file 1: Table S1. Ig levels range and median of the Low/Medium/High subcohorts. **Table S2.** The % of subjects with the same level of Ig for the three classes. **Table S3.** Markers of B cells in children homozygous for 3′RR1 hs1.2 allele. Additional file 2: Figure S1. Dot-plot of the data summarized in Table 1; hs1.2 alleles detected in each subject are on the x-axis, and Ig level detected in the same subject on the y-axis. Red lines represent the Low/Medium and Medium/High Ig-expression limits, as reported in Additional file 1: Table S1.
