# Supplementary material for: The change in Ig regulation from children to adults disconnects the correlation with the 3′RR hs1.2 polymorphism
Source: BMC Immunol. 2014 Nov 13;15:45. doi: 10.1186/s12865-014-0045-0 (PMC4234878; doi:10.1186/s12865-014-0045-0)
Supplement: Additional file 1: Table S1. — Ig levels range and median of the Low/Medium/High subcohorts. Table S2. The % of subjects with the same level of Ig for the three classes. Table S3. Markers of B cells in children homozygous for 3′RR1 hs1.2 allele. [file 12865_2014_45_MOESM1_ESM.docx]

**Table S1**

|  |  | **IgM** | | |  | **IgG** | | |  | **IgA** | | | **n.** |
| --- | --- | --- | --- | --- | --- | --- | --- | --- | --- | --- | --- | --- | --- |
|  |  | **LOW** | **MEDIUM** | **HIGH** |  | **LOW** | **MEDIUM** | **HIGH** |  | **LOW** | **MEDIUM** | **HIGH** |  |
| **CHILDREN** | **RANGE** (mg/dl) | 34 / 101 | 102 / 148 | 151 / 288 |  | 441 / 918 | 919 / 1230 | 1231 / 1983 |  | 15 / 88 | 89 / 136 | 137 / 295 | 190 |
|  | **MEDIAN** (mg/dl) | 90 | 110 | 210 |  | 867 | 1004 | 1554.5 |  | 50.5 | 111 | 160 |  |
|  | **n.** | 60 | 70 | 60 |  | 60 | 70 | 60 |  | 60 | 70 | 60 |  |
| **ADULTS** | **RANGE** (mg/dl) | 46.9 / 97.6 | 97.8 / 162 | 163 / 343 |  | 187 / 1095 | 1100 / 1350 | 1351 / 1865 |  | 62.5 / 148 | 149 / 216 | 219 / 444 | 340 |
|  | **MEDIAN** (mg/dl) | 74.8 | 126 | 204 |  | 994.5 | 1210 | 1460 |  | 118 | 181 | 267 |  |
|  | **n.** | 110 | 120 | 110 |  | 110 | 120 | 110 |  | 110 | 120 | 110 |  |

| **a Children** | | | | | | | | | |
| --- | --- | --- | --- | --- | --- | --- | --- | --- | --- |
|  | | **IgG** | | |  | **IgA** | | |  |
|  |  |  |  |  |  |  |  |  |  |
|  |  | **LOW** | **MEDIUM** | **HIGH** | **n.** | **LOW** | **MEDIUM** | **HIGH** | **n.** |
| **IgM** | **LOW** | 58% | 38% | 4% | 60 | 53% | 41% | 6% | 60 |
|  |  |  |  |  |  |  |  |  |  |
|  | **MEDIUM** | 32% | 56% | 12% | 70 | 40% | 47% | 13% | 70 |
|  |  |  |  |  |  |  |  |  |  |
|  | **HIGH** | 9% | 12% | 79% | 60 | 3% | 18% | 79% | 60 |
|  |  |  |  |  |  |  |  |  |  |

**Table S2: Percent of children (a) and adults (b) within the same Ig level group for Ig classes**

| **b Adults** | | | | | | | | | |
| --- | --- | --- | --- | --- | --- | --- | --- | --- | --- |
|  | | **IgG** | | |  | **IgA** | | |  |
|  |  |  |  |  |  |  |  |  |  |
|  |  | **LOW** | **MEDIUM** | **HIGH** | **n.** | **LOW** | **MEDIUM** | **HIGH** | **n.** |
| **IgM** | **LOW** | 29% | 38% | 33% | 110 | 33% | 34% | 33% | 110 |
|  |  |  |  |  |  |  |  |  |  |
|  | **MEDIUM** | 35% | 39% | 26% | 120 | 34% | 38% | 28% | 120 |
|  |  |  |  |  |  |  |  |  |  |
|  | **HIGH** | 33% | 28% | 39% | 110 | 30% | 34% | 36% | 110 |
|  |  |  |  |  |  |  |  |  |  |

**Table S3: comparison of B cell markers of homozygous children for *1 and *2 alleles at time 0**

|  | **Naive**  (% IgM-IgD+) | |  | **Marginal Zone** (%IgM high IgD low) | |  | **IgM Memory** (% IgM+IgD-) | |  | **Switched Memory**  (% IgM-IgD-) | |  | **CD19+** (cells/µl) | | **n.** |
| --- | --- | --- | --- | --- | --- | --- | --- | --- | --- | --- | --- | --- | --- | --- | --- |
|  |  |  |  |  |  |  |  |  |  |  |  |  |  |  |  |
|  | **MEDIA** | **MEDIAN** |  | **MEDIA** | **MEDIAN** |  | **MEDIA** | **MEDIAN** |  | **MEDIA** | **MEDIAN** |  | **MEDIA** | **MEDIAN** |  |
| **1/1** | 1.4 | 1.5 |  | 9.1 | 9.7 |  | 1.5 | 0.8 |  | 11.5 | 10.4 |  | 499 | 470 | 10 |
|  |  |  |  |  |  |  |  |  |  |  |  |  |  |  |  |
| **2/2** | 1.4 | 1.6 |  | 9.9 | 10.7 |  | 0.8 | 0.7 |  | 10.3 | 9.2 |  | 528 | 482 | 30 |
|  |  |  |  |  |  |  |  |  |  |  |  |  |  |  |  |
