# Supplementary figures and images for: The change in Ig regulation from children to adults disconnects the correlation with the 3′RR hs1.2 polymorphism
Source: BMC Immunol. 2014 Nov 13;15:45. doi: 10.1186/s12865-014-0045-0 (PMC4234878; doi:10.1186/s12865-014-0045-0)

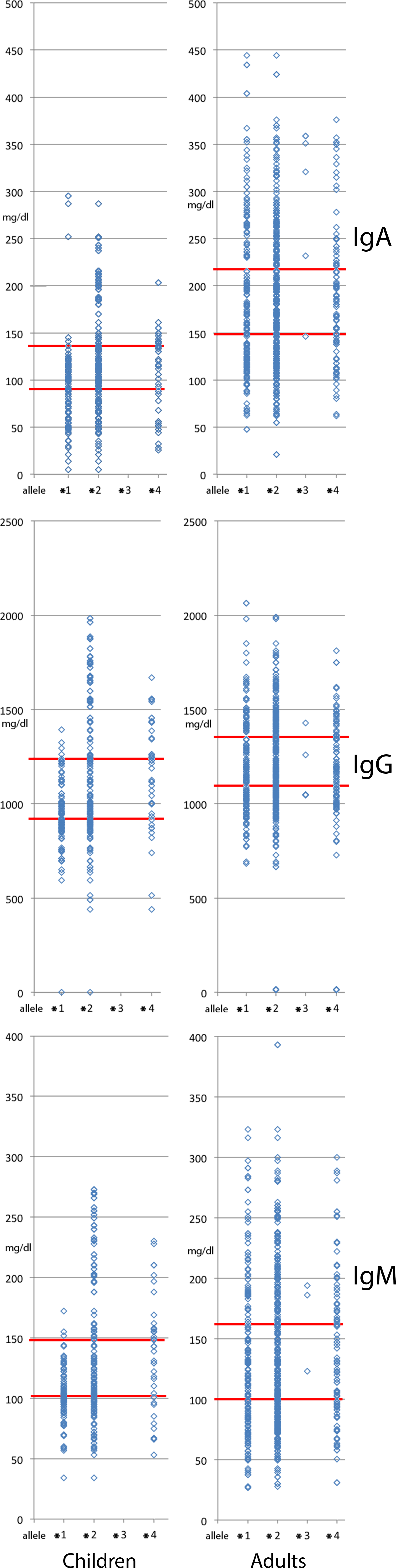

Supplement: Additional file 2: Figure S1. — Dot-plot of the data summarized in Table 1; hs1.2 alleles detected in each subject are on the x-axis, and Ig level detected in the same subject on the y-axis. Red lines represent the Low/Medium and Medium/High Ig-expression limits, as reported in Additional file 1: Table S1. [file 12865_2014_45_MOESM2_ESM.tiff]
